# The endogenous cannabinoid system modulates male sexual behavior expression

**DOI:** 10.3389/fnbeh.2023.1198077

**Published:** 2023-05-31

**Authors:** Gabriela Rodríguez-Manzo, Ana Canseco-Alba

**Affiliations:** ^1^Departamento de Farmacobiología, Centro de Investigación y de Estudios Avanzados (Cinvestav-Sede Sur), Ciudad de México, Mexico; ^2^Laboratorio de Fisiología de la Formación Reticular, Instituto Nacional de Neurología y Neurocirugía Manuel Velasco Suárez, Ciudad de México, Mexico

**Keywords:** endocannabinoids, male rat sexual behavior, sexual satiety, animal models of sexual dysfunction, mesolimbic circuit, sexual motivation, dopamine, brain plasticity

## Abstract

The endocannabinoid system (ECS) plays a key neuromodulatory role in the brain. Main features of endocannabinoids (eCBs) are that they are produced on demand, in response to enhanced neuronal activity, act as retrograde messengers, and participate in the induction of brain plasticity processes. Sexual activity is a motivated behavior and therefore, the mesolimbic dopaminergic system (MSL) plays a central role in the control of its appetitive component (drive to engage in copulation). In turn, copulation activates mesolimbic dopamine neurons and repeated copulation produces the continuous activation of the MSL system. Sustained sexual activity leads to the achievement of sexual satiety, which main outcome is the transient transformation of sexually active male rats into sexually inhibited animals. Thus, 24 h after copulation to satiety, the sexually satiated males exhibit a decreased sexual motivation and do not respond to the presence of a sexually receptive female with sexual activity. Interestingly, blockade of cannabinoid receptor 1 (CB1R) during the copulation to satiety process, interferes with both the appearance of the long-lasting sexual inhibition and the decrease in sexual motivation in the sexually satiated males. This effect is reproduced when blocking CB1R at the ventral tegmental area evidencing the involvement of MSL eCBs in the induction of this sexual inhibitory state. Here we review the available evidence regarding the effects of cannabinoids, including exogenously administered eCBs, on male rodent sexual behavior of both sexually competent animals and rat sub populations spontaneously showing copulatory deficits, considered useful to model some human male sexual dysfunctions. We also include the effects of cannabis preparations on human male sexual activity. Finally, we review the role played by the ECS in the control of male sexual behavior expression with the aid of the sexual satiety phenomenon. Sexual satiety appears as a suitable model for the study of the relationship between eCB signaling, MSL synaptic plasticity and the modulation of male sexual motivation under physiological conditions that might be useful for the understanding of MSL functioning, eCB-mediated plasticity and their relationship with motivational processes.

## 1. Introduction

The World Health Organization (WHO) recognizes that “sexuality is a central aspect of being human and encompasses sex, gender identities and roles, sexual orientation, eroticism, pleasure, intimacy and reproduction” and states, with robust research evidence, that sexual health positively correlates with mental health and life quality (Kismödi et al., 2017). In humans, sexuality is a multidimensional behavioral trait that includes biological, psychological, and sociocultural factors.

Since some of the brain circuits involved in different aspects of sexual behavior expression are highly conserved through evolution, the understanding of the central regulation of male sexual behavior derives substantially from preclinical studies mostly obtained in rodents. The use of animal models of human behavior reduces the practical and ethical issues that human studies represent. Also, establishing the role played by the different neurotransmitter systems, as well as the participation of specific brain areas in the control of male sexual activity, and other neurobiological bases of sexual behavior can only be studied through experimental models. Rodent male sexual behavior is considered to show good face validity, i.e., the consideration that the model is analogous or homologous to human behavior (Pfaus et al., 2003).

There are several neurotransmitters involved in the regulation of male rat sexual behavior and the actions of these neurotransmitters are highly regulated by other chemical messengers exerting modulatory effects. The endocannabinoid system (ECS) is one important neuromodulator of brain neurotransmitter actions. Here, we will discuss ongoing work investigating how endocannabinoid (eCB) signaling influences male sexual behavior (MSB) expression by modulating the activity of the mesolimbic dopaminergic system (MSL) and will review the effects of exogenously administered eCBs on the sexual behavior display of different male rat sexual sub populations, spontaneously showing copulatory deficits. Specifically, we will describe the sexual behavior of male rats and its the central regulation. We also summarize some of the models used for the study of different aspects of male sexual activity and the distinct sexual sub populations we can find in rats (Section “2. Male rat sexual behavior”). In the next section, we provide an overview of the ECS (Section “3. The endocannabinoid system”) and thereafter we review the available knowledge on cannabinoid effects on male sexual behavior, including the effects of Cannabis preparations on male human sexuality. We also review the involvement of the ECS in the control of MSB expression in sexually proficient animals (Section “4. Cannabinoid effects on male sexual activity”). Thereafter, we summarize the available data on a possible role of the ECS in the expression of MSB in sexually experienced rats (Section “5. Role of the ECS in the regulation of MSB expression”). We then expand the knowledge on this role of eCBs to a model of sexual inhibition: sexual satiety (Section “6. Endocannabinoids and the sexual satiety phenomenon”). To that aim we first describe the sexual satiety phenomenon and its relationship with the MSL system. We continue reviewing current knowledge on the role played by the ECS at the MSL circuit in the induction of brain plastic changes associated to the induction of this phenomenon. We conclude that sexual satiety is a model that allows the study of MSL system functioning, eCB-mediated plasticity, and its relationship with motivational processes under physiological conditions (Section “7. Concluding remarks”).

## 2. Male rat sexual behavior

### 2.1. Description

**FIGURE 1:**
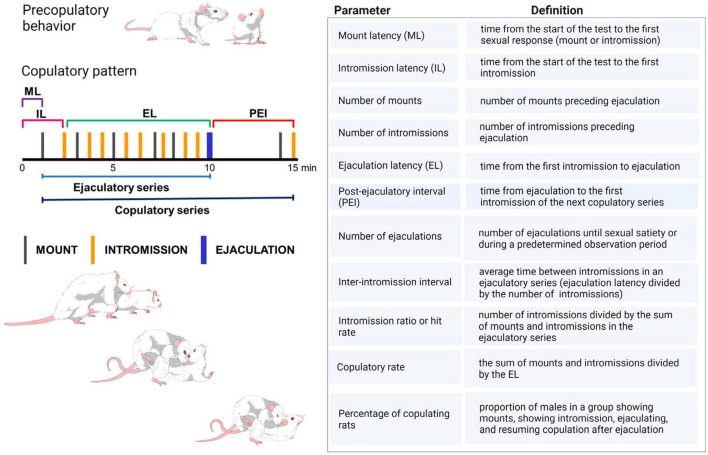
Copulatory pattern of the male rat. The male exhibits a series of mounts and intromissions until achieving ejaculation. During mounting the male lifts his forebody over the female hindquarters, clasping her flanks with its forepaws while keeping its hindlegs on the floor, and begins a series of rapid shallow movements of the pelvis without achieving penetration. Thereafter, the male dismounts the female gently. The intromission is a mount in which the male achieves erection and penetration, after which intravaginal pelvic thrusting is observed, followed by abrupt dismounting while jumping backward. The male usually grooms its genitals. Ejaculation is distinguishable by a longer-lasting intromission during which a deep pelvic thrust is observed that coincides with seminal emission. Thereafter, the male slowly dismounts the female and may elevate its forepaws. Ejaculation signals the end of an ejaculatory series, after which a short period of sexual refractoriness appears, the post-ejaculatory interval, which ends once the male restarts copulation with an intromission, completing a copulatory series. ML, mount latency; IL, intromission latency; EL, ejaculation latency; PEI, post-ejaculatory interval (Larsson, 1956; Sachs and Barfield, 1976; Dewsbury, 1979). Table shows the definition of the specific copulatory parameters utilized in the experimental analysis of male sexual behavior in rats. Created with BioRender.com.

Sexual behavior in male rats consists in the presentation of various sexually related anticipatory behaviors that include female anogenital investigation, sniffing and licking, as well as the emission of 50 kHz ultrasonic vocalizations. These behaviors are followed by the display of a highly stereotyped copulatory pattern. Male rat copulatory pattern is shaped by three distinct motor behaviors (Figure 1): (i) mounting behavior, during which the male puts its forepaws on the female’s flanks and shows pelvic thrusting, without accomplishing vaginal penile insertion; (ii) intromission behavior, consisting of a mount during which the male presents pelvic thrusting, penile erection, and attains intravaginal penile insertion and (iii) ejaculatory behavior, consisting of an intromission of longer duration during which seminal emission occurs (for a detailed description see Hull and Rodríguez-Manzo, 2017).

During sexual performance, the male rat presents several mounts and intromissions before achieving ejaculation. After ejaculating, male rats exhibit a transient period of sexual refractoriness, named the post-ejaculatory interval, which ends when they are able to renew sexual intercourse, indicated by the presentation of an intromission that initiates a new copulatory series. The copulatory responses displayed from the start of the first intromission until ejaculation constitute an ejaculatory series and, when the post-ejaculatory interval is included, it is named copulatory series (Figure 1).

Although innate, the copulatory pattern becomes more efficient and stable through sexual experience, in such a way that its temporal development (latencies to initiate sexual activity, to ejaculate and to resume sexual activity after ejaculating) and the stimulation needed to achieve ejaculation (number of pre-ejaculatory mounts and intromissions) become very similar among subjects, allowing the definition of efficient sexual activity through the establishment of baseline sexual measures. These stable basal copulatory measures serve as control parameters for comparisons with the effects of experimental manipulations on male rat sexual behavior. Thus, sexually experienced male rats, defined as those achieving ejaculation in less than 15 min, in at least three of five independent sexual training sessions, are used as control group. The most common sexual parameters used to evaluate male rat copulatory behavior are described in Figure 1.

### 2.2. Male sexual behavior physiological mechanisms

Studies on male rat sexual activity have made a distinction between its motivational and consummatory components (Beach, 1956). Sexual motivation results from the interaction of external sexual incentive stimuli and the animal’s capacity to respond to them and arouse, eliciting approach responses toward the potential sexual partner to enable copulation. Copulation itself represents the consummatory component of sexual behavior, which is shaped by three distinct sexual motor responses, i.e., mount, intromission and ejaculation. Though, the distinction between these two components is not straightforward as some overlapping of them exists (Pfaus, 1996; Ball and Balthazart, 2008). The participation of different mechanisms in the control of the motivational and the consummatory aspects of male sexual behavior was proposed, with specific neural systems underlying each of them (Everitt, 1990, 1995), among which the hypothalamic medial preoptic area (mPOA) and the MSL system have a prominent participation. The mPOA is a key integrative structure for male sexual behavior expression in all known vertebrate species (Hull and Rodríguez-Manzo, 2017). This brain region is involved mainly in the consummatory component of male rat sexual behavior i.e., sexual performance (Everitt, 1990), although its contribution to sexual motivation can be inferred from the diminished sexual motivation produced by mPOA lesions (Hull and Rodríguez-Manzo, 2017). In turn, the MSL system, constituted by the dopaminergic neurons arising from the ventral tegmental area (VTA) and ascending to the nucleus accumbens (NAcc) and medial prefrontal cortex (Ikemoto, 2007), plays a central role in the motivational component of male sexual behavior (Everitt, 1990; Melis and Argiolas, 1995), but it also participates in male sexual performance as evidenced by changes in NAcc neurons’ firing rate associated to sexual behavior execution (Matsumoto et al., 2012). Thus, overlapping of sexual motivation and performance is also evident in the roles played by each of these key neural systems in male sexual behavior expression.

### 2.3. Experimental models for the study of male sexual behavior

Male rodents share with men the capacity to engage in sexual activity continuously during their adult life. In addition, the brain structures, neural networks, and neurochemical control of male sexual activity appear to be common to both species (Georgiadis et al., 2012). Besides, drug actions on male rat copulatory behavior show a good predictive validity for its effects on human male sexual response. Therefore, the male rat is considered a good model for the study of the neurobiology and sexual pharmacology of the human male sexual response (Pfaus et al., 2003).

Male rat sexual behavior can be analyzed in the laboratory with different approximations, and a variety of models have been proposed for the study of specific aspects of male sexual activity including sexual arousal, sexual motivation and performance, and sexual inhibition. Some of the models addressing specific aspects of sexual activity are briefly described.

#### 2.3.1. Model of male sexual arousal

Erection is considered an indicator of sexual arousal in male rats and men, and the mechanisms involved in this sexual response are importantly shared between both species (Giuliano et al., 2010). The non-contact erection model is used to evaluate sexual arousal in rats. This model consists in exposing sexually experienced male rats to estrous females that are inaccessible but can be seen, heard, and smelled by the males. The sexual cues emitted by the estrous female evoke penile erections, considered to be of “psychogenic” origin, controlled at the central level, and to reflect sexual arousal (Sachs et al., 1994; Sachs, 2000; Nielsen et al., 2011).

#### 2.3.2. Model of male sexual motivation

**FIGURE 2:**
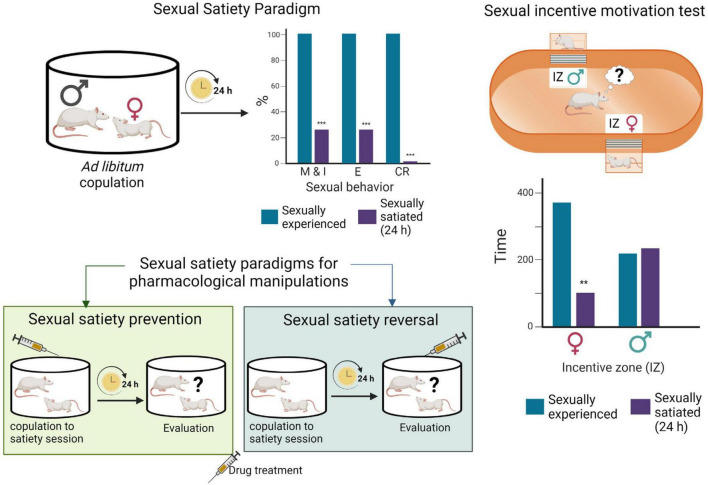
Sexual satiety paradigms. During *ad libitum* copulation, sexually experienced male rats ejaculate repeatedly (≈ 7 E) before reaching sexual satiety. Twenty-four hours later, the majority of these males (≈ 70%) will not show sexual activity in the presence of a receptive female. Drug treatments administered prior to the copulation to satiety session that prevent the sexual inhibitory state indicate the involvement of the targeted system in their induction, while treatments administered 24 h after copulation to satiety that reverse the established sexual inhibition, indicate the involvement of the targeted system in the maintenance of the sexual inhibitory state (Rodríguez-Manzo and Fernández-Guasti, 1994). Sexual incentive motivation test: The apparatus consists of an elliptic open field arena with two diagonally opposed windows, which are separated from the arena with wire mesh, and communicate each with a removable cage where the incentive animals are placed. Incentive zones are designated in the arena, in front of each window. The incentive animals are introduced into the cages and the experimental male put into the center of the arena and allowed to freely ambulate. Sexual incentive motivation is measured as the time spent by the male rat in the incentive zones (receptive female or male) of the field. Sexually experienced males will spend more time in the incentive zone of the sexually receptive female than in the incentive zone of the male. Sexually satiated males will spend the same amount of time in both incentive zones. Figure modified from Canseco-Alba and Rodríguez-Manzo (2019a). Created with BioRender.com.

Sexual incentive motivation, considered to model human sexual desire (Pfaus, 2009), is triggered in rats by unconditioned incentive sexual cues (odors of sexually receptive females) and can be measured with a non-conditioned test in which the sexual motivation of male rats, elicited by a sexually receptive female as opposed to the social motivation induced by another male rat, is assessed. In this test, the cumulative time that the experimental male spends in the social or sexual incentive zones, during 10 min, is considered indicative of the incentive motivation generated by each of the stimulus animals: a receptive female or a male (see Figure 2). Besides, specific copulatory measures are considered to reflect the level of sexual motivation. This is the case for both mount latency, which decrease is associated with an increased motivation, and for the display of a large number of mounts in animals that cannot achieve an erection, such as those with an anesthetized penis (Clark et al., 1984).

#### 2.3.3. Model of male sexual performance

The most common analysis to assess male rat sexual performance consists of the recording of sexual activity in sexually experienced male rats during a single copulatory series, however, it has been proposed that a more extended observation period (30 min), allowing the display of two to three successive copulatory series, offers a better panorama (Chan et al., 2010; Heijkoop et al., 2018). Sexually experienced male rats model an intact and optimal copulatory activity and are therefore useful for studying the neurobiology of male sexual behavior, to assess consummatory sexual parameters and to determine sexual inhibitory effects of experimental manipulations. This model is less useful for the establishment of sexual facilitatory effects of treatments (due to a ceiling effect) that can be better determined in sexually naïve rats (animals exposed for the first time to a sexually receptive female), which transiently show a deficient sexual performance that can still be improved.

#### 2.3.4. Model of male sexual inhibition

Inhibition of sexual behavior display in sexually competent male rats can be achieved through aversive conditioning (Pfaus et al., 2003), however, spontaneous sexual inhibition in sexually proficient male rats is only observed during sexual satiety. In this model, males are allowed to copulate without restriction with a same sexually receptive female, and they will typically ejaculate repeatedly before reaching a state of sexual inactivity named sexual satiety (Rodríguez-Manzo and Fernández-Guasti, 1994). Twenty-four hours later the satiated males are sexually inhibited, a condition that remains well established for 48 h, time after which it gradually disappears requiring a 15-day period of sexual rest for the animals to recover their initial ejaculatory capacity (Rodríguez-Manzo et al., 2011; Figure 2). The sexual inhibitory state during the first 48 h is expressed in the majority of the males by the absence of sexual activity in the presence of a sexually receptive female. In a small proportion of sexually satiated subjects, however, the sexual inhibition consists in the display of a single ejaculatory series, characterized by a deficient sexual performance, after which sexual activity is not resumed (Rodríguez-Manzo and Fernández-Guasti, 1994). Experimental manipulations can be oriented toward the study of the establishment of the sexual inhibitory state or be directed to analyze the expression of the long-lasting sexual inhibition (Figure 2).

### 2.4. Spontaneous models of sexual behavior deficiencies based on male rat sexual behavior variability

**FIGURE 3:**
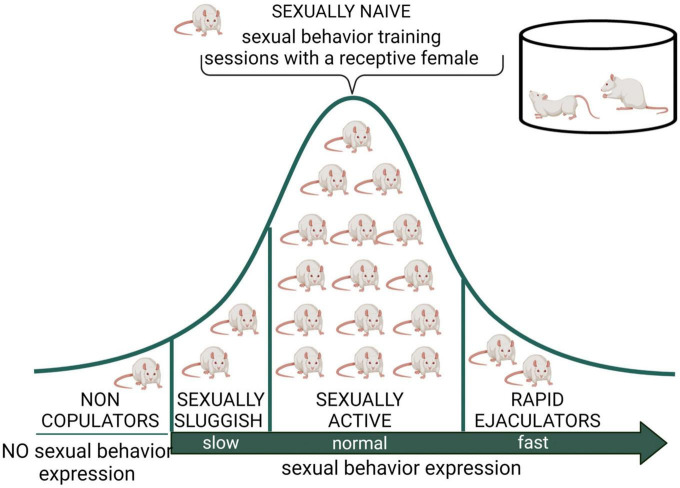
Distribution of male rat sexual populations. When sexually naïve male rats are exposed to a receptive female, most animals will display sexual behavior and its copulatory pattern will become stable after several training sessions. However, there is an intra-specific variability in this species, expressed in at least three subpopulations in terms of sexual behavior expression. The non-copulating male rat, representing around 20% of the population, is constituted by apparently normal and healthy animals that will not mate despite repeated exposure to sexually receptive females (Beach, 1942). The sexually sluggish male, whose incidence is highly variable but always present in rat populations, requires long latencies to initiate copulation and to achieve ejaculation (e.g., with ejaculation latencies longer than 30 min) that are not reduced by repeated sexual experience. The rapid ejaculators are animals that ejaculate in extremely short periods of time and therefore achieve 4–5 ejaculations in 30 min (normal males usually display a couple of ejaculations in that period) (Pattij et al., 2005). Non-copulating, sexually sluggish and rapid ejaculating male rats are naturally occurring sub populations that might model human sexual dysfunctions such as male hypoactive sexual desire disorder and asexuality, lifelong delayed ejaculation, and premature ejaculation, respectively. Created with BioRender.com.

Male rat’s natural biological variability permitted the identification of sexual sub populations, which could serve as preclinical models to study some human sexual dysfunctions. These sub populations include sexually inactive males (non-copulators), sexually sluggish males, and rapid ejaculators (Figure 3).

Sexually inactive male rats are sexually mature, healthy, apparently normal animals that do not mate when sexually receptive females are accessible, in spite of being repeatedly exposed to them. These animals are referred to as non-copulators (NC); they have intact genital responses (erection and ejaculation) (Stefanick and Davidson, 1987) and normal circulating sexual hormone levels (Whalen et al., 1961). The NC rat sub population could serve as a model for the study of the hypoactive sexual desire disorder (HSDD) found in clinical practice and described in the Diagnostic and Statistical Manual of Mental Disorders (DSM-5; American Psychiatric Association [APA], 2013).

Sexually sluggish male rats and rapid ejaculators have been considered to represent the two extremes of the Gaussian distribution of ejaculatory endophenotypes (Figure 3), proposed to be part of the biological variability in ejaculatory behavior (Pattij et al., 2005) present also in men (Waldinger, 2002). In one extreme of the distribution are the sexually sluggish rats, which are characterized by consistently requiring a long time to achieve ejaculation or not attaining ejaculation in a 30 min observation period. This prolonged ejaculation latency cannot be reduced by repeated sexual training. In the opposite extreme of this distribution are the rapid ejaculators, which are male rats that consistently show ejaculation latencies under 5 min, a feature that allows them to attain four to five ejaculations in a 30 min period of sexual interaction (Pattij et al., 2005). These two sub populations could represent models for the study of human life-long delayed ejaculation (DE) and premature ejaculation disorders, respectively, (Waldinger and Olivier, 2005).

## 3. The endocannabinoid system

All central nervous system functions, from reflexes to complex behaviors, ultimately result from the capability of neurons to electrochemically communicate with each other. Communication between neurons occurs at tiny gaps called synapses, where specialized parts of the two cells (i.e., the presynaptic and postsynaptic neuronal regions) come very close (a few nanometers) of one another, to allow for chemical transmission (Lovinger, 2008). A wide variety of molecules are known to act as neurotransmitters at the synapses, including serotonin, dopamine, noradrenaline, GABA, glutamate, and acetylcholine to name a few. Neurotransmission is an essential process that needs to be thoroughly regulated with the aid of other molecules acting as neuromodulators. Neuromodulation underlies the behavioral and neural circuit operation flexibility, ranging from short-term adjustments of neuron and synapse functioning to persistent long-term synaptic plasticity (Nadim and Bucher, 2014).

**FIGURE 4:**
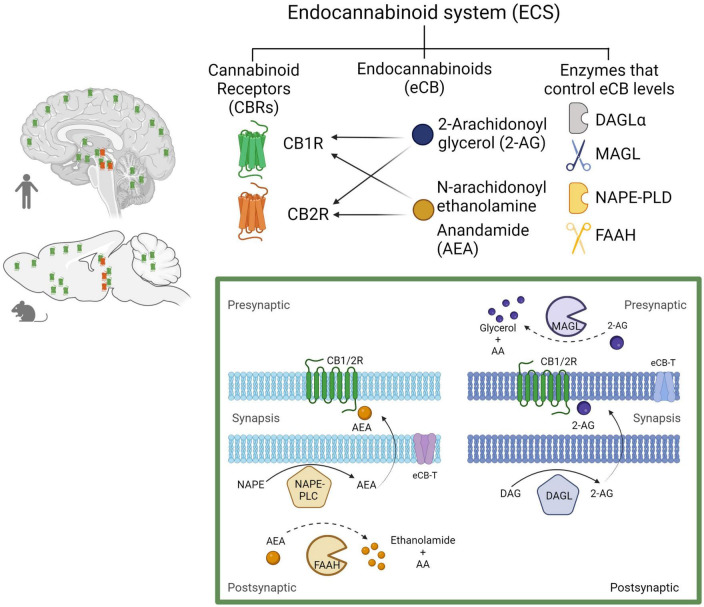
The endocannabinoid system. Composed by two Gi/o-coupled cannabinoid receptors (CB1R and CB2R), their endogenous ligands (2-AG and AEA), and the enzymes involved in their synthesis (DAGLα and NAPE-PLD) and degradation (MAGL and FAAH). In the human and rodent brains, the CB1R is highly expressed in neurons, while the CB2R is mainly expressed in microglial cells in discrete brain areas as represented (CB1R in green and CB2R in red). Biosynthesis and degradation of endocannabinoids: eCBs are synthetized from membrane phospholipid precursors. N-arachidonoylethanolamine (anandamide, AEA) is generated from its membrane precursor N-arachidonoyl phosphatidylethanolamine (NAPE) by a phospholipase D (NAPE–PLD). AEA is metabolized into ethanolamide and arachidonic acid (AA) by the action of the fatty acid amide hydrolase (FAAH) (Di Marzo et al., 1994). 2-AG is synthetized from membrane inositol phospholipids by a phospholipase C (PLC) producing diacylglycerol (DAG), which is hydrolyzed by the diacylglycerol lipase α (DAGLα). 2-AG degradation by monoacylglycerol lipase (MAGL) into glycerol and AA accounts for ≈ 85% of its breakdown (Sugiura et al., 1995). Created with BioRender.com.

The ECS has emerged as an important and widespread neuromodulatory system capable of regulating the levels and activity of most of the other neurotransmitter systems, when it is needed, providing immediate feedback. The ECS comprises cell membrane receptors (cannabinoid receptors) and their endogenous ligands (endocannabinoids) along with the enzymes and molecules required for their synthesis and degradation (Castillo et al., 2012; Figure 4).

### 3.1. CBR

**FIGURE 5:**
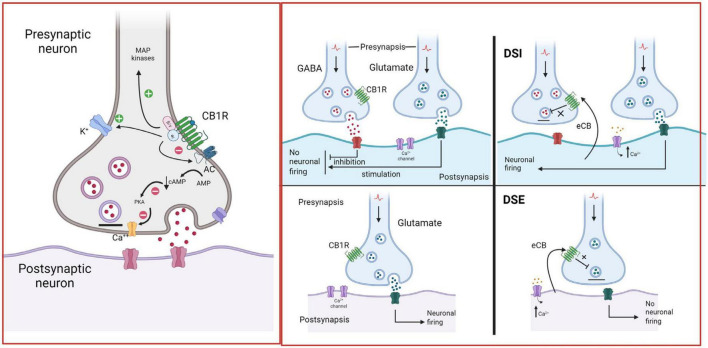
Endocannabinoid retrograde signaling. In the brain, CB1R are mainly located on presynaptic axon terminals, where their activation hyperpolarizes the terminal and decreases neurotransmitter release. Anandamide (AEA) and 2-arachidonoylglycerol (2-AG) are the primary endogenous ligands of CBR. AEA is a partial CB1R agonist, while 2-AG is a full CB1R agonist. eCBs are synthetized by postsynaptic neurons “on demand”, i.e., in response to heightened neuronal activity and increased intracellular Ca^2+^ concentrations. They diffuse through the postsynaptic membrane to the synaptic cleft, travel backward, and activate CB1R at the presynaptic terminals of adjacent neurons, suppressing neurotransmitter release through the short-term plasticity forms: depolarization-induced suppression of inhibition (DSI) at GABAergic synapses and depolarization-induced suppression of excitation (DSE) at glutamatergic synapses (Alger, 2002; Wilson and Nicoll, 2002; Melis, 2004). Created with BioRender.com.

The ECS includes two receptor subtypes known as cannabinoid receptor 1 (CB1R) and cannabinoid receptor 2 (CB2R). The CB1R is encoded by the *Cnr1* gene, and the protein consists of 472 amino acids, while the CB2R is encoded by the *Cnr2* gene, and the protein consists of 360 amino acids in humans. At the protein level, CB1R and CB2R have 44% sequence homology (Zou and Kumar, 2018). Both CB1R and CB2R are G protein-coupled receptors (GPCR’s) of the Gi/o class. As such, their activation inhibits adenylyl cyclase activity, reducing cAMP levels and protein kinase A (PKA) activity, block certain voltage-dependent calcium channels and activate several MAP kinase pathways, thereby regulating nuclear transcription factors (Howlett, 2005). In addition, CB1R activate inwardly rectifying potassium channels (GIRKs) (Guo and Ikeda, 2004; Figure 5).

At the central nervous system (CNS) CB1R are especially abundant in the cortex, basal ganglia, hippocampus, and cerebellum (Mackie, 2005). The great majority of CB1R can be found on axon terminals and pre-terminal axon segments, while sparing the active zone (Nyiri et al., 2005) and play a well-established role in CNS function and dysfunction (Durieux et al., 2022). By contrast, CB2R were found to play a central role in the immune system (Leleu-Chavain et al., 2013), while in the CNS, CB2R are predominantly expressed in microglia and only a few years ago were they identified on neurons, at the postsynaptic somatodendritic region, in discrete areas of the brain (Van Sickle et al., 2005; Onaivi et al., 2008). Now, there is some evidence for the involvement of CB2R in the modulation of neuronal function and behavior, and there is a growing research interest in their possible therapeutic implications (Dhopeshwarkar and Mackie, 2014; Kibret et al., 2022).

### 3.2. Endocannabinoids

Endocannabinoids, a contraction of the term endogenous cannabinoids (eCBs), are lipid signaling molecules, which have a structural similarity to molecules derived from the Cannabis plant, such as Δ^9^-tetrahydrocannabinol (Δ^9^-THC), hence its name. Both, eCBs and phytocannabinoids (molecules derived from Cannabis), bind to and activate cannabinoid receptors (CBR) (Fisar, 2009). N-arachidonoyl ethanolamine (anandamide, AEA) and 2-arachidonoylglycerol (2-AG) are the two best studied eCBs; both are derivatives of arachidonic acid and have similar chemical structures. However, 2-AG and AEA synthesis and breakdown follow distinct enzymatic pathways, suggesting that they are implicated in different physiological functions and pathophysiological processes (see Figure 4; Lu and Mackie, 2016).

The intrinsic efficacy of these eCBs for CBR also varies. 2-AG is a full agonist with moderate-to-low affinity for both CBR, whereas AEA is a high-affinity partial agonist of CB1R, and a very-low-efficacy agonist of the CB2R (Pertwee, 2015). Under certain conditions, AEA and 2-AG can exert their effects by activating other receptors, including other GPCRs like GPR55 (Pertwee et al., 2010), Transient Receptor Potential (TRP) channels, like vanilloid receptor TRPV1 (Lisboa and Guimarães, 2012), and nuclear Peroxisome Proliferator-Activated Receptors (PPARs), like PPARα and PPARγ (O’Sullivan, 2016). This non-CB1/2 receptor activity of eCBs accounts for some of the differential effects of certain cannabinoid agonists and pharmacological modulators of the eCB tone (Pacher et al., 2006).

Both AEA and 2-AG are synthetized “on demand” and released immediately, without storage in vesicles (Di Marzo et al., 1994; Sugiura et al., 1995; Fisar, 2009). Retrograde signaling is the major mechanism by which eCBs regulate synaptic function (Kano et al., 2009). eCBs are mobilized from postsynaptic neurons, travel backward across the synapse, bind to presynaptic CB1R, and suppress neurotransmitter release eliciting depression of excitatory and inhibitory synapses (see Figure 5; Alger, 2002). Evidence suggests that eCB-mediated effects at the synapse are more diverse (Castillo et al., 2012), because AEA is also a full agonist at TRPV1 channels (Smart et al., 2000), involving these channels in eCB signaling (De Petrocellis and Di Marzo, 2010). TRPV1 channels are expressed in the periphery, but also in the CNS, where they regulate synaptic plasticity and appear as promising pharmacological targets (Martins et al., 2014). Recent studies indicate that eCBs also signal via astrocytes to indirectly modulate presynaptic or postsynaptic function (Noriega-Prieto et al., 2023).

## 4. Cannabinoid effects on male sexual activity

### 4.1. Effects of phytocannabinoids and synthetic cannabinoids

The literature related to the effects of phytocannabinoids and exogenously administered eCBs on male sexual behavior (MSB) is scarce. In these studies, some data are more indirect than others, and in some cases, MSB analysis was not the main purpose of the referenced study and therefore the findings regarding MSB were not discussed in depth. In the next section we summarize and integrate the information available about this topic, considering that all data contribute to a certain extent to the understanding of the effects of targeting this neuromodulatory system on a complex behavior like MSB.

A lot of the knowledge about the behavioral effects of drugs acting as agonists at CBR comes from research on drugs of abuse and addiction. Many preclinical studies have been interested in characterizing the effects of Δ^9^-THC, since this molecule is recognized as the most important psychoactive compound of Cannabis preparations. We review here data on the sexual effects of CBR agonists in sexually active male rodents, which are summarized in Table 1. The first set of reviewed studies are those regarding the sexual effects of Cannabis preparations followed by those produced by the phytocannabinoid Δ^9^-THC; a third set summarizes the sexual effects of synthetic cannabinoids, and at the end those produced by exogenously administered eCBs (see Table 1).

**TABLE 1:** Studies investigating the acute effects of cannabinoid receptor agonists on male sexual behavior of sexually active rodents.

Species	Drug	Doses and administration route (latency)	Effects on MSB	References
**Cannabis preparations**				
Rat	Hashish resin	8 and 10 mg/kg, i.p.	↓% animals that engaged in copulation	Corcoran et al., 1974
Mouse	Tincture of cannabis (50 mg/ml ethanol). Content: 1.8 mg/ml Δ9-THC; 1.26 CBD; 0.58 CBN and 0.28 CBC	12.5 and 25 mg/kg, i.p. (−45 min)	12.5 mg/kg: ↓ frequency and duration of M and MA. 25 mg/kg: ↓ all sexual behavior responses	Cutler et al., 1975; Cutler and Mackintosh, 1984
Mouse	Hashish extract Content: 40%; Δ9-THC 45% CBD; 9% CBN and 6% other CBs	20 mg/kg, p.o. (−90 min)	↓ All sexual responses (general sedation). No sexual activity	Sieber et al., 1981
Mouse	Hashish extract Content: 40% Δ9-THC; 45% CBD; 9% CBN and 6% other CBs	20 mg/kg, p.o. (−90 min)	General sedation. In this experiment ♀ and ♂ were treated. ↓%M to 0	Frischknecht et al., 1982
Rat	Cannabis flowers	200 and 400 mg, vaporized for 10 min	No changes	Mondino et al., 2019
**Phytocannabinoids**				
Rat	Δ9-THC	2 and 3 mg/kg, i.p.	↑ latencies (ML, EL, and PEI) Dose-dependent ↓ in% of rats that reached E	Merari et al., 1973
Rat	Δ9-THC	0.025 mg/kg, i.p.	↓%M and %E ↓ competition for ♀ in estrus	Uyeno, 1976
Mouse	Δ9-THC	50 and 100 mg/kg, p.o. (−4 h)	Complete suppression of copulation; pre-copulatory behaviors (genital sniffing and grooming) were preserved	Dalterio et al., 1978
Rat	Δ9-THC	5 mg/kg, p.o. (−30 min)	↓%M and %I ↑ ML, IL	Murphy et al., 1994
**Synthetic cannabinoids**				
Rat	HU-210	0.025, 0.05, 0.1 mg/kg, i.p. (−40 min)	0.025 mg/kg: no effect 0.05 mg/kg: ↑ EL 0.1 mg/kg: ↓%M and %I (no E)	Ferrari et al., 2000
**Endocannabinoids (eCB)**				
Rat	AEA	0.5, 1, 2, 4 mg/kg, i.p.	1 mg/kg: ↑ #E/30 min 2 and 4 mg/kg: ↑ ML, ↑ IL; ↑ #I	Martinez-Gonzalez et al., 2004
Rat	AEA	0.1−10 mg/kg, i.p.	0.3 and 1.0 mg/kg: ↓ IL, ↓ EL, ↓ #I, ↑ #E/1 h 3.0 and 10 mg/kg: ↑ IL, ↑ EL, ↓ #E/1 h	Canseco-Alba and Rodríguez-Manzo, 2014; Rodríguez-Manzo and Canseco-Alba, 2015b
Rat	2-AG	0.03−3.0 mg/kg, i.p.	No changes	Canseco-Alba and Rodríguez-Manzo, 2019a

CBs, cannabinoids; Δ9-THC, tetrahydrocannabinol; CBD, cannabidiol; CBN, cannabinol; CBC, cannabichromene; AEA, anandamide; 2-AG, 2-arachidonoylglycerol; i.p., intraperitoneal; p.o., oral. Percentage of animals showing mount attempts: %MA; mounts: %M; intromissions: %I; ejaculation: %E. Sexual parameters: ML, mount latency; IL, intromission latency; EL, ejaculation latency; #M, number of mounts; #I, number of intromissions, #E/time, number of ejaculations observed during a particular period (30 min or 1 h).

Early studies with Cannabis preparations (Cutler et al., 1975; Sieber et al., 1980; Frischknecht et al., 1982; Cutler and Mackintosh, 1984) evaluated MSB as part of a broader study on their effects on overall social behavior in mice. Although the use of such preparations hinders the dissection of the effects of specific cannabinoids, they allow to conclude that the pharmacological activation of CBR with this mixture of compounds induces a dose-dependent inhibition of MSB (Corcoran et al., 1974; Cutler et al., 1975; Cutler and Mackintosh, 1984). Though, the Cannabis doses used in those studies also caused sedation, i.e., the animals exhibited an increased immobility, probably masking more subtle sexual effects that could have been observed with lower doses. Notwithstanding, in a recent study evaluating the sexual effects of lower doses of a Cannabis preparation delivered by vaporization, MSB was not altered, suggesting that Cannabis preparations not necessarily exert inhibitory actions on copulation (Mondino et al., 2019; Table 1, section a).

In humans, Cannabis consumption has a bidirectional effect on sexual activity. Low, acute doses may enhance human sexual functioning, specifically increasing sexual desire and satisfaction in some subjects, while large doses may produce negative effects on sexual functioning such as a lack of interest in sexual activity, erectile dysfunction, and inhibited orgasm, affecting sexual motivation (Abel, 1981; Balon, 2017). A paper from the group of Vicenzo Micale reviews the subjective reports of Cannabis users on different aspects of male sexuality (Androvicova et al., 2017). In Table 2, some representative studies of this topic are summarized, with emphasis in the studies reporting on sexual motivation and arousal. It is important to mention that several of these studies either did not find any effects of Cannabis (Smith et al., 2010) or report a general positive sexual outcome (Halikas et al., 1982; Wiebe and Just, 2019; Bhambhvani et al., 2020; Shiff et al., 2021) on male sexual self-reported behavior, while only some of them report negative sexual effects of its consumption (Aldemir et al., 2017) that seemed to be related to high Cannabis doses consumption (Koff, 1974; Chopra and Jandu, 1976). The different outcomes in those papers might be related with the multifactorial nature of human sexual behavior in the interpretation of the self-perceived Cannabis sexual effects as discussed in the classic paper of Sydney Cohen (1982).

**TABLE 2:** Studies analyzing the effects of Cannabis consumption on sexual behavior in men.

Target population	Method or instrument	Result	References
College students (345)	e-mail survey (cannabis use and sexuality)	Increase in sexual desire and enjoyment following the use of marijuana. As dosage increases, the tendency toward an increase in sexual desire decreases	Koff, 1974
Chronic marijuana users (*n* = 275)	Intense interviews	Low cannabis doses seem to act as an aphrodisiac. Performance and enjoyment are enhanced under its influence. Moderate and high cannabis doses, still induce desire but interferes with sexual performance.	Chopra and Jandu, 1976
Marijuana users (*n* = 50)	Interview	Users felt that marijuana acted as an aphrodisiac, but only about 9% rated the effect as strong; 81% reported increased feelings of sexual pleasure and satisfaction with marijuana use.	Halikas et al., 1982
8,656 Australians	Computer-assisted telephone survey	No association between frequency of cannabis use and sexual desire. Frequent cannabis use is related with changes in orgasm timing (delayed or too soon)	Smith et al., 2010
Patients with cannabis use disorder (*n* = 47)	International index of erectile function (IIEF)	Cannabis use is associated with erectile and orgasmic dysfunction in males. No differences in Sexual desire score or Sexual satisfaction score	Aldemir et al., 2017
People with experience using cannabis during sex (*n* = 216)	Online questionnaire	Participants reported that cannabis is related with an increased desire for sex and heightened sexual satisfaction. Also, an increased penile erectile function/hardness, increased sensitivity to touch and intensity of orgasm. In general, positive outcomes	Wiebe and Just, 2019
Adults who visited a cannabis dispensary (*n* = 325)	International index of erectile function (IIEF)	Frequency-response relationship between cannabis use and sexual function. Increased use associated with an overall increased IIEF score, intercourse satisfaction domain, and overall satisfaction domain.	Bhambhvani et al., 2020
Sexual and gender minority men (SGM)	In-depth, semi-structured interviews	Participants reported an instrumental use of cannabis to alleviate and address symptoms of mental health (e.g., depression, post-traumatic experiences) and describe adverse effects of cannabis use on their mental health, including feelings of paranoia.	Parent et al., 2021
Patients from academic center andrology clinic (*n* = 993)	Questionnaires sexual health inventory for men (SHIM)	Cannabis users had a higher mean SHIM and higher sexual frequency compared to non-users	Shiff et al., 2021

Another group of preclinical studies evaluated the specific effects of the phytocannabinoid Δ^9^-THC allowing a more direct interpretation of cannabinoid sexual effects. Acute Δ^9^-THC administration (Table 1, section b) had inhibitory effects on MSB reflected as increases in the temporal parameters of copulation, i.e., ML, IL, EL, and PEI, but not in the number of M or I. High doses of Δ^9^-THC reduced the proportion of animals showing the different sexual behavior responses (Merari et al., 1973; Ferrari et al., 2000) or significantly reduced the number of males that engaged in sexual behavior (Dalterio et al., 1978). Coinciding with the results obtained in the early studies with cannabinoid mixtures, Δ^9^-THC dose-dependently inhibited MSB, mainly affecting the temporal sexual parameters. However, due to the high Δ^9^-THC doses employed in those studies, it must be considered that general locomotion could have been altered and, therefore, unspecific drug effects might underly the observed sexual inhibitory effects.

The same dose-dependent inhibition of MSB was observed with the synthetic cannabinoid HU-210 (Table 1, section c). In this case, the lower dose lacked effects, but the intermediate dose significantly increased the EL, considered to be an inhibitory effect. Finally, with the highest dose tested, the percentage of animals showing sexual behavior was reduced and those copulating did not reach ejaculation (Ferrari et al., 2000). It is important to consider that HU-210 is a synthetic cannabinoid, sevenfold more potent than Δ^9^-THC at neuronal CBR (Devane et al., 1992).

As mentioned above, in one of the early studies (Sieber et al., 1981) acute administration of 20 mg/kg Δ^9^-THC, contained in a hashish extract, induced sedation (an increase in immobility time) in male mice and, as a consequence, no sexual behavior was observed. In this case and taking into account that the temporal sexual parameters were the most affected by CB1R agonists, it is possible that the observation period (10 min) was not sufficient to detect changes in sexual behavior. This is a probable explanation since in another set of experiments performed by the same research group, it was reported that even though during the observation period (20 min) mounting behavior was not observed, the next day both vehicle- and drug-treated females showed seminal plugs, evidencing that the males eventually showed sexual activity (Frischknecht et al., 1982).

### 4.2. Effects of endocannabinoids

The MSB results obtained after the exogenous administration of eCBs (Table 1, section d) evidence that the two most studied eCBs play a neuromodulatory role and exert differential effects on MSB. Thus, a wide range of 2-AG doses did not modify MSB of sexually experienced animals (Canseco-Alba and Rodríguez-Manzo, 2019a). By contrast, AEA produced dose-dependent biphasic effects on MSB, with low doses facilitating and high doses inhibiting different sexual parameters of sexually proficient male rats (Martinez-Gonzalez et al., 2004; Canseco-Alba and Rodríguez-Manzo, 2014). The facilitatory effects of low AEA doses were mediated by CB1R, while the inhibitory effects of higher AEA doses involved TRPV1 activation (Rodríguez-Manzo and Canseco-Alba, 2015b).

The described acute sexual effects of CBR activation are relevant to demonstrate that eCBs modulate MSB expression, most likely through short-term plasticity processes. The effects of repeated CBR stimulation on MSB can be recognized from studies exploring the effects of sub chronic and chronic administration of CBR agonists. The main objective of those studies was to understand the sexual consequences of long-term intake of Cannabis preparations in humans. The next set of reviewed studies are those exploring the sexual effects of sub chronic or chronic treatment with CBR agonists in rodents, which are shown in Table 3. Sub chronic treatment refers here to repeated administration over a relatively short period, while chronic treatment refers to repeated administration for a large portion of the lifespan. These two approaches contribute to the understanding of the long-term effects of CBR agonists on MSB.

**TABLE 3:** Studies investigating the effects of repeated administration of cannabinoid receptor agonists on male sexual behavior of sexually active rodents.

Species	Drug	Doses, route of administration and treatment schedule	Effects on MSB	References
**Sub-chronic treatment with CBR agonists**				
Mouse	Hashish extract	20 mg/kg THC, p.o. sub-chronic (3 days, 1X/d) tested daily	1st day: sedation no sexual activity 2nd day: tolerance to the sedative effects. ↓%AM, %M, and %I but not ≠ from control 3rd day: ↑%AM, %M, and %I	Sieber et al., 1981
Rat	HU-210	0.025−0.1 mg/kg, i.p. sub-chronic (14 days, 1X/d) tests: 7th day and 14th day	0.025 mg/kg: ↑ trend in ML, IL, and EL; ↓% CS (7th day > 14th day) 0.05 mg/kg: ↑ ML, IL, EL, and PEI and ↓ #M, #I; ↓% CS (7th day > 14th day) 0.1 mg/kg: ↓%CS (7th day > 14th day)	Ferrari et al., 2000
Rat	HU-210	0.1 mg/kg, i.p. sub-chronic (10 days, 1X/d) test: day 11	Trend toward ↑ in all temporal parameters. ↓ I and E frequency; test was run after the last administration (−30 min)	Riebe et al., 2010
**Chronic administration of CBR agonists**				
Rat	Δ9-THC Synthetic CME (16% Δ9-THC)	0.5, 1.5, and 5 mg/kg, p.o. chronic (60 days, 1X/d) test: day 61	No differences in mating with any dose or compound evaluated by fertility indices (no specific parameters were evaluated). Administration from PND 40 onward	Wright et al., 1976
Mouse	Hashish extract (“*idem”*)	20 mg/kg, p.o. chronic [3X/week for 12 weeks (36 doses)] tests: 26th or 27th day of treatment	Decrease in social investigation, specially sexually motivated behavior, whereas non-social activities were significantly increased. The next morning all drug-treated females had vaginal plugs (evidencing sexual interaction) Treatment from weaning onward. (♀ and ♂ treated)	Frischknecht et al., 1982
Mouse		50 mg/kg, p.o. chronic [3X/week for 7 weeks (21 doses)] tests: 3rd and 7th week of treatment	3rd week: ↑ ML and IL 7th week: ↑ ML	Dalterio, 1980
Rat		10 mg/kg, p.o. chronic (30 days, 1X/d) test: day 30th	Loss of libido (failure to show mounting behavior with a non-receptive female in a 15 min session)	Dhawan and Sharma, 2003

CBR, cannabinoid receptor; CME, crude marijuana extract; PND, postnatal day; i.p., intraperitoneal; p.o., oral. Percentage of animals showing attempted mounts: %AM; mounts: %M; intromissions: %I. Sexual parameters: ML, mount latency; IL, intromission latency; EL, ejaculation latency; PEI, post-ejaculatory interval; CS, copulatory series (i.e., the males resumed copulation after ejaculation).

The first important finding with the sub chronic treatment with CBR agonists is the development of tolerance in different animal responses. In one of the early studies on the sexual effects of sub chronic Δ^9^-THC, it was reported that the first administration of 20 mg/kg Δ^9^-THC from a hashish extract induced sedation in male mice and, as a consequence, no sexual behavior was observed. However, the animals developed tolerance to these sedative effects by the second day of treatment, and therefore in contrast to the previous day, some animals displayed MSB, which was indistinguishable from the sexual performance of control animals. The only parameter affected was what the authors called “attempted mount” (apparently an unsuccessful mount), which they considered indicative of sexual motivation. More interesting, by the third day of treatment, in addition to tolerance to the sedative effects, the number of mice showing “attempted mount” was significantly larger than in the vehicle-treated group, suggesting tolerance development to Δ^9^-THC sexual effects. Also, the number of animals showing mounts and intromissions was slightly increased in Δ^9^-THC-treated males in comparison with control mice (Sieber et al., 1981; Table 3 section a). This represents, to the best of our knowledge, the first report on the facilitative effects of a CBR agonist on MSB.

Another study reported the development of tolerance to the sexual effects of the synthetic cannabinoid agonist HU-210, in a sub chronic protocol of daily administration along 14 days. In this study, sexual impairments were observed at all HU-210 doses tested, which became more evident halfway of the treatment (day 7) than during the last evaluation (day 14) (Ferrari et al., 2000). Another study with the same compound found that after 10 days of daily administration, there was only a trend toward an increase in the duration of the temporal parameters, however, a significant decrease in the number of intromissions and ejaculations was observed (Riebe et al., 2010).

The reviewed studies evaluating the sexual effects of chronically administered CBR agonists were mainly directed to determine their endocrine and reproductive consequences, thus, MSB evaluation was limited (Table 3, section b). The most recent of these studies reported a “loss of libido” in male rats, interpreted from the failure of males to mount a non-receptive female, after 30 days of daily administration of 10 mg/kg Δ^9^-THC (Dhawan and Sharma, 2003). A 12-week treatment with hashish extract in mice (20 mg/kg Δ^9^-THC; 3X/week) decreased sexually motivated behavior and social investigation (Frischknecht et al., 1982). Another study found that treatment with 50 mg/kg Δ^9^-THC (3X/week) for 3 weeks significantly increased ML and IL, however, after 7 weeks of treatment ML was the only parameter still augmented (Dalterio, 1980). This result suggests that Δ^9^-THC-induced sexual inhibitory effects are also susceptible to tolerance development. Two other studies on the chronic effects of Cannabis preparations in rats, using different doses of synthetic Δ^9^-THC or its equivalent with crude marijuana extract (0.5, 1.5, and 5 mg/kg) along 60 days, either did not assess MSB specific parameters or did not find differences with the control group (Wright et al., 1976). All these studies were performed in sexually competent animals.

As mentioned earlier, there are specific male rat sub populations in terms of MSB expression, spontaneously showing specific sexual deficiencies (Figure 3). The effects of CBR agonists, mostly those of the eCB AEA, have been explored in these sub populations (see Table 4).

**TABLE 4:** Studies investigating the effects of cannabinoid receptor agonists on sexual behavior of male rat sexual sub populations.

Sub population	Drug	Doses and route of administration	Effects on MSB (acute)	References
Sexually inactive (SI) rats	HU-210	0.25, 0.5 mg/kg, i.p.	0.5 mg/kg: ↓contact latency and genital exploration time with the receptive female	Ferrari et al., 2000
Sexually naïve rats	AEA (eCB)	0.3 mg/kg, i.p.	↑%E ↓ IL, #M, #I during the 1st exposure to the receptive female	Rodríguez-Manzo and Canseco-Alba, 2015b
Sluggish rats	AEA (eCB)	0.1−3.0 mg/kg, i.p.	0.3 and 1.0 mg/kg: ↓#I 0.3, 1.0 and 3.0 mg/kg: ↓ EL	Rodríguez-Manzo and Canseco-Alba, 2015a
Non-copulating rats	AEA (eCB)	0.03−1.0 mg/kg, i.p.	0.1 and 0.3 mg/kg: ↑% rats showing M, I, E, and CR	Canseco-Alba and Rodríguez-Manzo, 2019b

eCB, endocannabinoids; AEA, anandamide; i.p., intraperitoneal. Percentage of animals showing mounts: %M; intromissions: %I; ejaculation: %E; copulation resumption after ejaculation: %CR. Sexual parameters: IL, intromission latency; EL, ejaculation latency; #M, number of mounts; #I, number of intromissions.

Anandamide exerted differential effects on these sub populations but interestingly improving the specific sexual deficiencies of each of them. Thus, in rats previously classified as NC this eCB induced the display of sexual behavior in a long-lasting manner (at least for 14 days), suggesting that AEA acute administration was able to transform the animals’ sexual condition from inactive into sexually active (Canseco-Alba and Rodríguez-Manzo, 2013). In sexually sluggish males, AEA reduced the number of intromissions and EL, lowering their ejaculatory threshold although only for the duration of AEA effects, in contrast to the long-term effect produced in the NC males. The effects of HU-210 were also tested in a population of undefined sexually inactive rats and was found to decrease the latencies to contact and to initiate genital investigation of the female but without inducing sexual activity (Ferrari et al., 2000). Although the mechanisms through which the exogenous administration of eCBs modifies sexual behavior expression in these sub populations are not completely understood, it has been demonstrated that AEA actions were mediated by CB1R and that the NC males required a sexual interaction under AEA effects to be transformed into sexually active animals (Rodríguez-Manzo and Canseco-Alba, 2015a; Canseco-Alba and Rodríguez-Manzo, 2019b). AEA also facilitated sexual behavior performance in sexually naïve rats during their first sexual encounter, when usually copulation is disorganized and inefficient due to inexperience (Rodríguez-Manzo and Canseco-Alba, 2015b).

## 5. Role of the ECS in the regulation of male sexual behavior expression

The most direct strategy to understand the role played by the ECS in MSB expression is its manipulation under physiological conditions with the aim of pharmacological tools. One approach is to interfere with eCB’s putative actions during sexual activity by blocking CBR with specific antagonists; if this manipulation modifies a specific sexual response, a physiological role for eCBs in that behavior can be inferred. Another approach is to increase the eCB tone by inhibiting the reuptake and/or degradation of specific eCBs (Table 5).

**TABLE 5:** Studies investigating the effects of blocking eCB actions at CB1R or enhancing eCB levels on sexual behavior expression of sexually active male rats.

Drug	Mechanism of action	Dose and administration route (latency)	Effects on MSB	References
AM251	CB1R antagonist	1, 2 and 5 mg/kg, i.p. (−90 min)	5 mg/kg: ↓ #I; ↓ EL	Gorzalka et al., 2008
AM251	CB1R antagonist	0.1, 0.3, 1.0, and 3.0 mg/kg, i.p.	1 and 3 mg/kg: ↓#M, ↓EL, ↑#E/1h 3 mg/kg: ↓IL	Canseco-Alba and Rodríguez-Manzo, 2014
AM404	Inhibitor of AEA reuptake and FAAH inhibitor	1, 2 and 5 mg/kg, i.p. (−90 min)	5 mg/kg: ↑ IL	Gorzalka et al., 2008
URB597	FAAH inhibitor	0.1, 0.3 and 0.5 mg/kg, i.p.	No effect	Gorzalka et al., 2008
SR 141716A (Rimonabant)	CB1R antagonist	0.5, 1 and 2 μg/rat intra-PVN	Induced spontaneous penile erection	Melis et al., 2004

eCB, endocannabinoid; CB1R, cannabinoid receptor 1; FAAH, fatty acid amide hydrolase; AEA, anandamide; i.p., intraperitoneal; intra-PVN, infused into the hypothalamic paraventricular nucleus. Sexual parameters: ML, mount latency; IL, intromission latency; EL, ejaculation latency; #M, number of mounts; #I, number of intromissions, #E, number of ejaculations.

The available data show that antagonism of CB1R with low AM251 doses lacked sexual effects (Gorzalka et al., 2008; Canseco-Alba and Rodríguez-Manzo, 2014), similar to the results obtained with the indirect increase of endogenous AEA levels by inhibiting its hydrolysis with the FAAH inhibitor URB597 or by blocking its reuptake and degradation with AM404, which almost lacked MSB effects (only an increase in the IL was observed after the highest dose tested) (Gorzalka et al., 2008). Notwithstanding, facilitatory effects on MSB of sexually experienced male rats have been documented after higher AM251 doses, suggesting that eCBs could play an inhibitory role on MSB expression (Gorzalka et al., 2008; Canseco-Alba and Rodríguez-Manzo, 2014). However, this interpretation is not straightforward, because AM251 also behaves as an inverse agonist and, therefore, could produce effects opposite to the agonist actions of endogenous ligands (Tallett et al., 2007).

Infusion of the CB1R antagonist SR 141716A (Rimonabant) into the paraventricular hypothalamic nucleus (PVN) has been found to induce spontaneous penile erection, an effect apparently mediated by the activation of oxytocinergic transmission, which is considered to indicate sexual arousal. However, infusion of CB1R agonists into the PVN lacked effects on this sexual reflex (Melis et al., 2004, 2006).

Together, data suggest that the ECS plays no predominant role in the modulation of MSB of sexually proficient animals but rather becomes relevant when its functioning is altered, as in NC and sexually sluggish animals, which condition most probably involves neurotransmitter imbalances. This puts forward the potentiality of targeting the ECS in the search for new therapeutic agents for the treatment of sexual dysfunctions, as suggested for AEA in the management of lifelong delayed ejaculation (Fenner, 2015).

Main triggers for eCB synthesis and release are enhanced neuronal activity and the increase of intracellular Ca^2+^ concentrations that might result from this augmented neuronal activity or from the direct activation of metabotropic receptors coupled to phospholipase C, like group I metabotropic glutamate receptors (mGluR1 and mGluR5) (Covey and Yocky, 2021). It is considered that one of the main functions of eCBs is to avoid excitotoxicity and return cell functioning to a homeostatic balance (Diana and Marty, 2004). In the following section we will provide evidence for the release of eCBs in the mesolimbic system (MSL), as a result of intense sexual activity during copulation to satiety, and their participation in the induction of the long-lasting sexual inhibitory state of the sexually satiated males and in the modulation of MSL dopamine neuron activity, which might represent control mechanisms to counteract excessive stimulation.

## 6. The ECS and the sexual satiety phenomenon

### 6.1. Sexual satiety

Sexually experienced male rats allowed to copulate without restriction with a single sexually receptive female will ejaculate repeatedly (seven successive ejaculations in average) until becoming sexually satiated (Beach and Jordan, 1956; Rodríguez-Manzo and Fernández-Guasti, 1994). Sexual satiety is characterized by the emergence of a long-lasting sexual inhibitory state resulting from intense copulation within a short period (around 2.5 h). This naturally induced sexual inhibition has been postulated to have an adaptive nature, representing a mechanism to guard individuals against threatening situations when engaging in excessive sexual activity or to avoid the lowering of the available sperm store due to excessive ejaculation (Bancroft, 1999).

This sexual inhibitory state remains well established for 48 h after copulation to satiety and is expressed in the majority of the males by the absence of sexual activity in the presence of a sexually receptive female (Rodríguez-Manzo and Fernández-Guasti, 1994). The absence of sexual response during the 48 h that follow repeated ejaculation, is a remarkable phenomenon given the instinctive nature of sexual responding in the presence of a sexually receptive female and the proved sexual proficiency of the animals prior to the copulation to satiety session. The sexual inhibition of the satiated males gradually fades away evidencing its reversible nature (Rodríguez-Manzo et al., 2011).

Twenty-four hours after copulation to satiety, the well-established sexual inhibitory state can be pharmacologically reversed by different drug treatments (see Table 6) that have as a common feature the ability to interact with the dopaminergic system. At the same time point, i.e., 24 h after the sexual satiety session, male rats exhibit physiological modifications in addition to the sexual inhibition, when compared to non-satiated males. These include the loss of the sexual facilitatory effect produced by electrical stimulation of the main brain regions involved in the control of copulatory behavior, like the mPOA (Rodríguez-Manzo et al., 2000), the VTA (Rodríguez-Manzo and Pellicer, 2007) and the NAcc (Rodríguez-Manzo and Pellicer, 2010); a decreased sexual motivation (Canseco-Alba and Rodríguez-Manzo, 2019a; Guadarrama-Bazante and Rodríguez-Manzo, 2019) and the manifestation of hypersensitivity to several drugs. This hypersensitivity consists in the appearance of specific drug effects, described for high doses, at lower doses not eliciting them in non-satiated males and this response has been observed with compounds acting at different neurotransmitter systems. Among them we can mention the noradrenergic (yohimbine), endogenous opioid (naloxone and naltrexone), serotonergic (8-OH-DPAT) and dopaminergic (haloperidol) systems (Rodríguez-Manzo and Fernández-Guasti, 1994, 1995a,b; Rodríguez-Manzo, 1999). Besides, the sexually satiated rats exhibit sensitized responses to the antidepressant desipramine (Martínez-Mota et al., 2005) and to methamphetamine-induced hyperlocomotion and rewarding effects (Violante-Soria et al., 2023). Together, these data suggest the occurrence of brain plasticity processes as a result of copulation to satiety. Several lines of evidence suggest that these plasticity processes might take place at the mesolimbic system with the participation of the ECS.

**TABLE 6:** Pharmacological treatments reversing the sexual inhibition of sexually satiated male rats.

Neurotransmitter system	Drug (doses) and administration route	Mechanism of action	References
GABAergic system	Bicuculline (0.01−0.3 mg/kg), i.p. Bicuculline (50 ng/rat), intra-VTA	GABA-A receptor antagonist	Rodríguez-Manzo and Canseco-Alba, 2017
Glutamatergic system	Ketamine (0.01−0.3 mg/kg), i.p.	Non-competitive NMDA receptor antagonist	Rodríguez-Manzo, 2015
	CNQX (0.001−0.003 mg/kg), i.p.	AMPA receptor antagonist	
	MPEP (0.03 mg/kg), i.p.	mGluR5 receptor antagonist	
Noradrenergic system	Yohimbine (2.0−4.0 mg/kg), i.p.	α2-adrenoceptor antagonist	Rodríguez-Manzo and Fernández-Guasti, 1994
Serotonergic system	8-OH-DPAT (0.25−0.5 mg/kg) i.p.	5-HT1A receptor agonist	Rodríguez-Manzo and Fernández-Guasti, 1994
Dopaminergic system	Apomorphine (25−100 μg/kg), i.p.	Non-selective DA receptor agonist	Guadarrama-Bazante et al., 2014
	SKF38393 (3.0 mg/kg), i.p.	D1-like DA receptor agonist	
	Apomorphine (0.6−6.0 μg/rat), intra-NAcc	Non-selective DA receptor agonist	Guadarrama-Bazante and Rodríguez-Manzo, 2019
	Quinpirole (0.3−1.0 μg/rat), intra-NAcc Quinpirole (0.3 μg/rat), intra-mPOA	D2-like DA receptor agonist	
Opioidergic system	Naloxone (3.0 mg/kg), i.p. Naltrexone (0.2 mg/kg), i.p.	μ and δ opioid receptor antagonists	Rodríguez-Manzo and Fernández-Guasti, 1995a
	Naltrexone (0.3 μg/rat), intra-VTA	μ and δ opioid receptor antagonist	Garduño-Gutiérrez et al., 2013
Endocannabinoid system	AEA (0.1−0.3 mg/kg), i.p.	CBR agonist	Canseco-Alba and Rodríguez-Manzo, 2014
	AEA (0.01−0.03 μg/rat), intra-VTA	CBR agonist	Canseco-Alba and Rodríguez-Manzo, 2016
	2-AG (0.1−3.0 mg/kg), i.p.	CBR agonist	Canseco-Alba and Rodríguez-Manzo, 2019a

i.p., intraperitoneal; intra-VTA, infused into the ventral tegmental area; intra-NAcc, infused into the nucleus accumbens; intra-mPOA, infused into the hypothalamic medial preoptic area.

### 6.2. Sexual satiety, endocannabinoids, and the mesolimbic circuit

The MSL circuit is essential for the processing of natural rewarding behaviors (Kelley and Berridge, 2002; Salamone et al., 2016), like sexual behavior. MSL dopamine plays a central role in the mediation of motivation, behavioral activation, reward processing and learning (Salamone and Correa, 2012; Leventhal et al., 2014; Yang et al., 2018). In the presence of reward-associated stimuli, the dopaminergic neurons of the MSL system are activated and change their firing pattern (Floresco et al., 2003; Grace et al., 2007) causing a substantial increase in NAcc dopamine release (Wightman and Robinson, 2002; Berke, 2018). Changes in NAcc dopamine concentrations alter reward processing and motivated behaviors.

**FIGURE 6:**
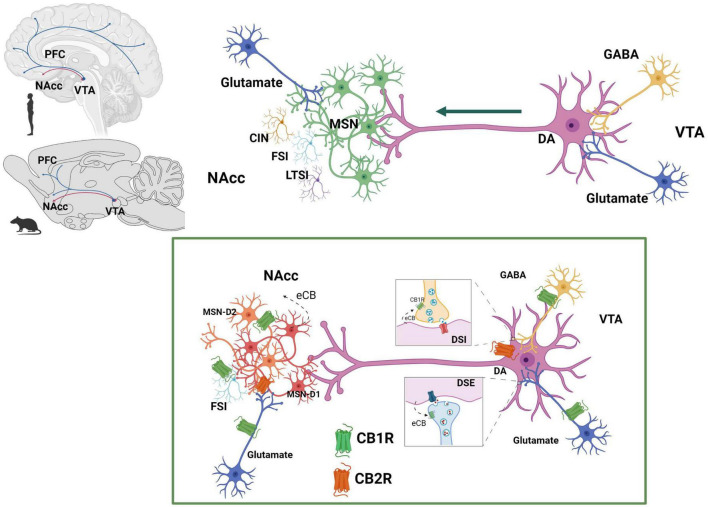
Endocannabinoid system at the mesolimbic circuit. The dopaminergic neurons of the mesolimbic (MSL) system originate in the ventral tegmental area (VTA) and project to the nucleus accumbens (NAcc) and the prefrontal cortex (PFC) (Ikemoto, 2007). Dopaminergic neurons’ activity is regulated by GABAergic and glutamatergic inputs at the VTA. NAcc neuronal population includes predominantly GABAergic median spiny projection neurons (MSN) (90–95%) and three different types of interneurons: cholinergic interneurons (CIN), GABAergic fast spiking interneurons (FSI) and GABAergic persistent low threshold spiking interneurons (LTSI). Within the MSL system, eCBs are released from dopamine neurons in the VTA and from MSN in the NAcc. CB1R are expressed on GABAergic (Szabo et al., 2002) and glutamatergic (Melis, 2004) axon terminals of the VTA and on afferent glutamatergic axon terminals (Robbe et al., 2001) and GABAergic FSI (Winters et al., 2012) of the NAcc. CB2R have been reported to be expressed on dopaminergic neurons at the VTA (Zhang et al., 2014, 2017) as well as on MSN-D1 neurons at the NAcc (Zhang et al., 2022). Created with BioRender.com.

The activity of the dopaminergic neurons of the MSL system is regulated at the VTA, where the ECS components i.e., receptors and ligands as well as eCB synthetizing and degrading enzymes, are expressed (Lupica et al., 2004; Figure 6).

**FIGURE 7:**
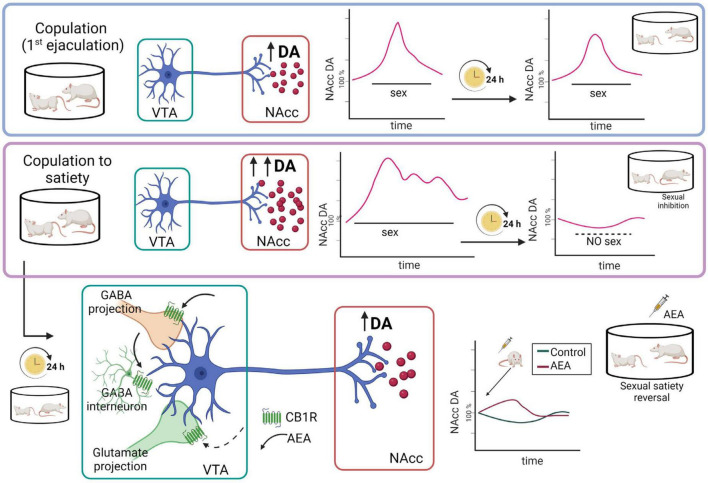
Representation of the hypothetic mechanistic explanation for the AEA-induced reversal of the sexual inhibition that results from copulation until satiety. Although copulation to one ejaculation produces an increase in nucleus accumbens (NAcc) dopamine (DA) concentrations similar to the one produced by copulation to satiety, in the latter case the increased dopamine levels remain elevated for a longer period and, 24 h later, NAcc dopamine basal levels are significantly reduced, coinciding with the sexual inhibition of the sexually satiated rats. AEA administration to the sexually satiated rats produces a slight increase in NAcc dopamine levels (red line) that coincides with the display of copulation indicating sexual satiety reversal (Canseco-Alba et al., 2022). AEA might have activated CB1R inhibiting GABAergic transmission, which could be responsible for the increase in dopamine release at the NAcc. Created with BioRender.com.

There is sound evidence for the activation of the MSL system by male rat sexual activity, indicated by the increase in NAcc dopamine extracellular concentrations (Pfaus et al., 1990; Damsma et al., 1992; Robinson et al., 2001), augmented c-Fos expression in VTA dopamine neurons (Balfour et al., 2004), and enhanced VTA neuronal firing (Hernandez-Gonzalez et al., 1997). It has been demonstrated that NAcc dopamine levels remain elevated during the process of copulation until satiety (Fiorino et al., 1997; Canseco-Alba et al., 2022), implying that the MSL system is continuously activated (Figure 7). Since enhanced midbrain dopamine neuron activity triggers the synthesis and release of eCBs from their cell bodies in the VTA (Lupica and Riegel, 2005), the constant dopaminergic neuronal activation that takes place during copulation to satiety might release eCBs at the MSL system that contribute to the establishment of the long-lasting sexual inhibition, characteristic of the sexually satiated rats. A first approach to test this hypothesis was to block CB1R with an antagonist during the copulation to satiety session. We found that the establishment of the sexual inhibition in males that copulated to satiety was prevented when eCB actions were hindered by blocking CB1R, demonstrating that eCBs participate in the induction of the sexual satiety phenomenon (Canseco-Alba and Rodríguez-Manzo, 2014). In addition, we demonstrated that this CB1R blockade also prevented the emergence of the characteristic drug hypersensitivity of sexually satiated rats (González-Morales and Rodríguez-Manzo, 2020). Since these results were established with the systemic administration of AM251, the next step was to determine the involvement of the MSL system in both effects. The prevention of both phenomena was successfully replicated when infusing the CB1R antagonist directly into the VTA (González-Morales and Rodríguez-Manzo, 2020). These data support the notion that one mechanism for the induction of the long-lasting sexual inhibition and drug hypersensitivity phenomena of males that copulate to satiety involves the release of eCBs and the activation of CB1R at the VTA. The demonstration of an increase in CB1R internalization in VTA neurons of male rats that copulated to satiety the day before, reinforce this notion (Rodríguez-Manzo et al., 2021). It is important to mention that CB1R are expressed on different MSL neuronal subpopulations, both at the VTA and NAcc as illustrated and documented in Figure 6.

Microdialysis studies showed that MSL dopamine transmission is altered 24 h after copulation to satiety, since NAcc dopamine basal concentrations were significantly diminished in sexually satiated males when compared to non-satiated rats. Moreover, exposure of the sexually satiated males at that time point to a receptive female did not induce the typical increase in NAcc dopamine release but rather dopamine concentrations decreased further (Figure 7). This blunted dopamine response coincided not only with the sexual inhibitory state (Canseco-Alba et al., 2022) but also with a decreased sexual motivation (Canseco-Alba and Rodríguez-Manzo, 2019a) in the sexually satiated males (see Figure 2). As mentioned before, low AEA doses reverse the sexual inhibition that characterizes sexual satiety both when systemically administered and after its intra-VTA infusion (Canseco-Alba and Rodríguez-Manzo, 2014, 2016). Interestingly, the systemic administration of an AEA dose reversing sexual satiety, induced a slight increase in the diminished NAcc dopamine concentrations of sexually satiated rats that coincided with the reinstatement of their ability to copulate with the receptive female (Figure 7). This result suggests that NAcc dopamine concentrations have to attain a threshold for animals to be capable of responding to the rewarding stimulus that the receptive female represents. In support of this hypothesis, dopamine agonists directly infused into the NAcc also reverse sexual satiety (Guadarrama-Bazante and Rodríguez-Manzo, 2019). Furthermore, the eCB-induced reversal of sexual satiety has been shown to involve the dopaminergic system and, interestingly, AEA and 2-AG interact each with a different dopamine receptor family; while the AEA/dopamine interaction involves D1-like receptors, the 2-AG/dopamine interaction involves D2-like receptors (Canseco-Alba and Rodríguez-Manzo, 2019a). One mechanism through which AEA might have induced the reversal of sexual satiety is the activation of CB1R at VTA GABAergic nerve endings, inhibiting GABAergic transmission, which could result in the increase in NAcc dopamine release (see Figure 7). The reversal of sexual satiety produced by the blockade of VTA GABA-A receptors supports this possibility (Rodríguez-Manzo and Canseco-Alba, 2017). Notwithstanding, the contribution of blocking CB1R located on VTA glutamatergic nerve endings to AEA-induced satiety reversal, by a yet not identified mechanism, cannot be discarded since antagonism of AMPA, NMDA and mGluR5 glutamatergic receptors also reverses sexual satiety (Rodríguez-Manzo, 2015).

### 6.3. Endocannabinoids and mesolimbic plasticity in the sexual satiety phenomenon

The activity of the dopaminergic neurons of the MSL system is regulated at the VTA, a brain region mainly composed of dopamine neurons (55−65%) and GABA interneurons (30%) but also containing a small proportion of glutamatergic neurons (5%) (Yamaguchi et al., 2007; Nair-Roberts et al., 2008; Dobi et al., 2010; Morales and Root, 2014). In this brain area dopamine neuron activity is predominantly regulated by GABAergic and glutamatergic inputs. On the one side, local GABAergic interneurons together with GABA projections, arising from the rostromedial tegmental nucleus (Matsui and Williams, 2011; Bouarab et al., 2019), impinge VTA dopamine cell bodies exerting a tonic inhibitory influence on their activity. On the other side, glutamatergic projections arising from the medial prefrontal cortex (Tzschentke, 2001), the pedunculopontine tegmental nucleus (PPT) and the laterodorsal tegmental nucleus (LDT) (Clements and Grant, 1990) are activated by behaviorally relevant stimuli releasing glutamate at the VTA, which contributes to change dopamine neuron firing pattern to its phasic mode by activating NMDA receptors located on dopamine cell bodies (Floresco et al., 2003).

Dopamine release at the NAcc depends both on dopamine neuron activity (Floresco et al., 2003) and the local control of dopamine terminal release (Mohebi et al., 2019). The typical NAcc dopamine response to a rewarding stimulus, including a sexually receptive female, consists of an increase in NAcc dopamine release. The hypodopaminergic NAcc basal tone of the sexually satiated rats during the sexual inhibitory period, together with the blunted response of dopaminergic neurons in the presence of a sexually receptive female could be the result of the activation of regulatory mechanisms of NAcc dopamine terminal release (Nolan et al., 2020) and/or of synaptic plasticity phenomena inducing changes in dopamine neuron functioning.

The main mechanisms reducing or inhibiting NAcc dopamine terminal release involve: (i) The D2 autoreceptor-mediated feedback (Beaulieu and Gainetdinov, 2011) inhibiting vesicular dopamine release, decreasing dopamine synthesis, and increasing dopamine uptake, in addition to the D2-autoreceptor-mediated inhibition of dopamine neuron excitability and modulation of its firing rate that is produced at the somatodendritic region (Ford, 2014); (ii) the membrane dopamine transporter (DAT) mediated synaptic dopamine clearance (Egaña et al., 2009); (iii) the activation of GABA-B (Pitman et al., 2014) and metabotropic glutamate group I (mGlu I) heteroreceptors on dopamine terminals, which activation inhibits dopamine release (Zhang and Sulzer, 2003); (iv) the GABA-A receptor mediated indirect suppression of NAcc dopamine release (Brodnik et al., 2019) and (v) the nicotinic acetylcholine heteroreceptor-mediated inhibition of dopamine release that takes place during heightened dopaminergic neuronal activity (Zhang and Sulzer, 2004). Several of these mechanisms could play a role in the reduced NAcc dopamine basal levels and the blunted dopamine neuron response to the rewarding stimulus that have been identified in the sexually satiated males (Canseco-Alba et al., 2022).

An additional level of control of mesolimbic dopamine neuron activity is exerted by the eCB release in the VTA that emerges during prolonged or heightened dopamine neuron discharge (Riegel and Lupica, 2004). The released eCBs bind to presynaptic CB1R, located on glutamatergic (Melis, 2004) and GABAergic (Szabo et al., 2002) nerve endings inhibiting neurotransmitter release at glutamatergic terminals through the DSE (Kreitzer and Regehr, 2001) and at GABAergic terminals through the DSI (Wilson and Nicoll, 2002) short-term plasticity phenomena (see Figure 5), further modulating the firing activity of mesolimbic dopamine neurons at the VTA. eCBs might also activate the more recently identified CB2R on VTA dopamine neurons, inhibiting dopaminergic neuronal firing (Zhang et al., 2014, 2017).

Endocannabinoid-mediated regulation of MSL dopamine neuron activity appears particularly relevant to the sexual satiety-associated changes in MSL system functioning, as these lipid messengers play a role in the induction of the long-lasting physiological and behavioral changes of the sexually satiated rats (González-Morales and Rodríguez-Manzo, 2020).

Investigating the possible mechanisms mediating these eCB actions have been the matter of our latest interest. Based on the evidence showing that eCBs induce long-term synaptic plasticity phenomena at VTA GABAergic (Chevaleyre and Castillo, 2003) and glutamatergic synapses (Gerdeman et al., 2002) and on the well-known participation of VTA glutamatergic transmission in long-term plasticity phenomena (Xin et al., 2016), as well as in the induction of dopamine neuron burst firing in response to relevant and rewarding stimuli (Floresco et al., 2003), we explored the possibility that synaptic plasticity phenomena involving glutamate and eCBs could be triggered as a result of copulation to satiety.

The VTA neurons that are activated during copulation, identified by c-Fos protein expression, receive glutamatergic inputs (Balfour et al., 2006). Thus, synaptic plasticity phenomena could be triggered at these glutamatergic synapses with the participation of the eCBs that are released in the VTA during copulation to satiety to modify dopamine neuron functioning and contributing to the induction of the long-lasting sexual inhibition and drug hypersensitivity of sexually satiated male rats.

Glutamatergic plasticity involves changes in the density and subunit composition of α-amino-3-hydroxy-5-methylisoxazole-4-propionate (AMPA) and N-methyl-D-aspartate (NMDA) receptors (Song and Huganir, 2002; Paoletti et al., 2013). We recently demonstrated the occurrence of such changes in AMPA and NMDA glutamate receptors of the VTA of the sexually satiated rats 24 h after copulation (Rodríguez-Manzo et al., 2021). Interestingly, most of these changes were related to eCB-mediated CB1R activation. Thus, a decrease in AMPAR density was found in the satiated rats that was prevented when CB1R were blocked with the antagonist AM251 during the development of sexual satiety. An increase in the proportion of AMPAR containing the GluA2 subunit was also found in the sexually satiated males, which was eCB-independent (Rodríguez-Manzo et al., 2021).

GluA2-containing AMPAR are impermeable to Ca^2+^, while GluA2-lacking AMPARs are Ca^2+^- permeable. The repeated activation of GluA2-lacking AMPAR triggers a long-lasting switch in their composition to GluA2-containing AMPARs (Liu and Cull-Candy, 2000). This switch in AMPAR composition has been associated to long-term depression (LTD) of excitatory synapses (Mameli et al., 2007). Thus, the increase in GluA2 subunit expression in the sexually satiated rats indicates a reduced Ca^2+^ signaling at AMPAR.

A decrease in the expression of the GluN2A NMDAR subunit, concomitant to an increase in GluN2B NMDAR subunit expression was also found in the VTA of sexually satiated males, and both changes were eCB-dependent (Rodríguez-Manzo et al., 2021). NMDAR containing the GluN2A subunit are characteristic of active synapses, while NMDAR containing the GluN2B subunit are found at inactive synapses (Ehlers, 2003). Thus, the increased expression of GluN2B NMDAR subunit suggests an increase in inactive synapses. These changes in the expression of AMPAR and NMDAR subunit composition indicate a reduction in the activity of VTA glutamatergic synapses in the sexually satiated rats, which is compatible with their decreased dopamine neuron activity, reflected both in the lower NAcc dopamine basal concentrations and the blunted dopamine response to a stimulus normally eliciting an important increase in NAcc dopamine release (Canseco-Alba et al., 2022). Other plastic changes recently reported in the sexually satiated male rats are a decrease in DAT density and an increase in D1-like dopamine receptor expression in the NAcc (Violante-Soria et al., 2023).

These MSL molecular changes constitute the first identified synaptic plasticity events associated to sexual satiety that could be involved in the behavioral and physiological modifications that characterize the sexually satiated male rats.

### 6.4. Sexual satiety as a model for the understanding of mesolimbic functioning and eCB-mediated plasticity

The fact that most changes in the molecular composition of VTA NMDA, and AMPA receptors detected in the sexually satiated rats were mediated by eCBs, together with their involvement in the induction of the long-lasting physiological and behavioral changes, suggests that eCB-dependent plasticity might constitute one of the mechanisms operating at the MSL system as a result of its intense activation, to counteract excessive stimulation. Thus, these mechanisms might be relevant for the induction of the alterations in MSL system functioning associated with the motivational deficits that accompany neuropsychiatric disorders. The central role played by the ECS in the regulation of MSL system working, poses it as a target for the development of potential therapeutic agents for the treatment of motivational deficiencies, including those associated to drug abuse.

The sexual satiety model allowed the identification of distinct MSL system adaptations that coincide with a decreased sexual motivation and the inhibition of an instinctive motivated behavior in the presence of its triggering stimulus. These adaptations include changes in the NAcc dopamine levels and in dopamine release in response to a rewarding stimulus, revealing a change in dopamine neuron activity; molecular changes in VTA glutamatergic receptors, indicative of a decreased excitatory transmission on dopamine neuron activity; and changes in the expression of dopamine associated proteins in the NAcc of the satiated rats.

Based on these data, the sexual satiety model appears as an important instrument for understanding the functioning of the MSL system, its relationship with motivational processes, and the identification of the control mechanisms that operate during its intense activation.

This model offers the following possibilities: (i) to follow the transient, but long-lasting, change in the functioning of the MSL system triggered by its repeated activation by a natural stimulus under physiological conditions; (ii) to identify the mechanisms involved in changing MSL dopamine neuron function, and (iii) the impact of such changes on motivational states. Moreover, the reversible nature of the long-lasting physiological changes that accompany the sexual satiety phenomenon offers the possibility to detect unidentified mechanisms capable of restoring MSL system functioning.

## 7. Concluding remarks

We can conclude from the reviewed data. On the one side, that exogenously administered eCBs modulate MSB expression. Although AEA and 2-AG exert their sexual effects through the activation of a same receptor, the CB1R, their pharmacological profile differs. Thus, AEA but not 2-AG effects on MSB are biphasic, with low doses facilitating and high doses inhibiting or losing their facilitative effects on copulation. The modulatory actions of eCBs on MSB become more evident in males with sexual deficiencies, in which exogenously administered eCBs restore those deficiencies either transiently or in a long-lasting manner. On the other side, eCBs play a physiological role in the control of MSB expression in animals that copulate to satiety, through the induction of plastic changes at the MSL system that could represent a protective mechanism against the excessive activation produced by heightened natural rewarding stimulation. The sexual satiety phenomenon appears as a natural model for the study of MSL system functioning and its relationship with motivational processes. The knowledge obtained from the analysis of the sexual satiety phenomenon might shed light on different aspects of the neurobiology of MSB in men, as well as on the MSL circuit mechanisms underlying human motivational functions and dysfunctions associated with neuropsychiatric disorders.

## Author contributions

Both authors listed have made a substantial, direct, and intellectual contribution to the work, and approved it for publication.
